# Supplementary material for: Research on emission reduction investment strategies for low carbon technology enterprises
Source: PLoS One. 2025 Oct 27;20(10):e0324669. doi: 10.1371/journal.pone.0324669 (PMC12558561; doi:10.1371/journal.pone.0324669)
Supplement: S2 Fig — (DOCX) [file pone.0324669.s002.docx]

% Parameter settings

epsilon = 3; % Demand elasticity parameter

theta = 0.2; % Tax rate

I = 1064; % Investment amount

eta = 0.4; % Cost coefficient

e0 = 1.8; % Initial efficiency

lambda_val = 0.05; % Technical coefficient (using lambda_val to avoid conflict with MATLAB function)

Q = 1.7; % Efficiency parameter

P0 = 2; % Initial price

M = 31; % Market size

r0 = 0.05; % Base discount rate

mu0 = 0.2; % Base growth rate

% Initialization calculations

k = 20:10:80; % X-axis: Output quantity Q (range from 0.2 to 1)

sigma_vals = [0.2, 0.4, 0.6]; % Volatility values for three curves

line_styles = {'-', 'o-', '*-'}; % Line styles: circle, dot, asterisk

colors = lines(3); % Color scheme

figure; hold on;

% Iterate through different volatilities

for i = 1:length(sigma_vals)

sigma = sigma_vals(i);

r = r0 + sigma;

mu = mu0 - (sigma^2)/2;

factor1 = epsilon / (epsilon - 1); % ε/(ε-1) term

factor2 = (r - mu); % (r-μ) term

% Calculate PT(Q) function

A = (1 - theta) .* I ./ (eta .* e0 .* Q); % Term A

B = (k .* e0 .* Q .* lambda_val .* eta .* P0 .* Q .* M) ./ (r .* eta .* e0 .* Q); % Term B

PT = factor1 .* (A - B) .* factor2;

% Plot curves

plot(k, PT, line_styles{i}, 'Color', colors(i,:), ...

'LineWidth', 1.5, 'MarkerSize', 8, ...

'DisplayName', ['Volatility ', num2str(sigma)]);

end

h_legend = legend('Volatility 0.2 ','Volatility 0.4 ' ,'Volatility 0.6' ,'FontName', 'Times New Roman', 'FontSize', 12);

% For MATLAB versions prior to R2019b, use the following command to remove legend border

set(h_legend, 'Box', 'on');

set(h_legend, 'FontSize', 22); % You can adjust this value as needed

% Set font properties for axes and labels

set(gca, 'FontName', 'Times New Roman', 'FontSize', 22);

xlabel('Carbon tax rate ({\itk})', 'FontName', 'Times New Roman', 'FontSize', 22); % X-axis label

ylabel('Optimal investment carbon price ({\itP_t})', 'FontName', 'Times New Roman', 'FontSize', 22); % Y-axis label

% Remove right and top borders

% Bold X-axis and Y-axis lines

ax = gca;

ax.XAxis.LineWidth = 1; % Set X-axis line thickness

ax.YAxis.LineWidth = 1; % Set Y-axis line thickness

set(gca, 'Box', 'on')
