# Supplementary material for: Research on emission reduction investment strategies for low carbon technology enterprises
Source: PLoS One. 2025 Oct 27;20(10):e0324669. doi: 10.1371/journal.pone.0324669 (PMC12558561; doi:10.1371/journal.pone.0324669)
Supplement: Attachment — Submitted filename: Review of PONE-D-25-22903.pdf [file pone.0324669.s005.pdf]

Journal: PLOS ONE
Manuscript No.: **PONE-D-25-22903**
Title: Research on Emission Reduction Investment Strategies for Small and Medium-sized Enterprises under Low Carbon Technologies

The research developed the study models for Emission Reduction Investment Strategies for Small and Medium-sized Enterprises using Low-Carbon Technologies. The research evaluated the critical factors that simultaneously supported sustainable infrastructure investments. The evaluation is compared with the conventional method to show the superiority of the proposed plan. In addition, the proposed model was implemented on some case studies to verify its practical effectiveness. However, we have included some comments to enhance the quality of the manuscript:

1. As the author explains the weaknesses of several conventional methods, the author needs to explain several fundamental weaknesses of the proposed plan in terms of context of use, time, and environment. Besides, the authors can explore more about the advantages and disadvantages of the proposed method
2. All figures still need to be improved to make it easier to observe and read.
3. The paper is too long. The writing can be made shorter, more concise, and clearer.
4. Further, the author can address some potential detailed future research issues in the conclusion section.
