# Supplementary material for: Research on emission reduction investment strategies for low carbon technology enterprises
Source: PLoS One. 2025 Oct 27;20(10):e0324669. doi: 10.1371/journal.pone.0324669 (PMC12558561; doi:10.1371/journal.pone.0324669)
Supplement: Attachment — Submitted filename: Response to Reviewers.docx [file pone.0324669.s007.docx]

We sincerely thank the editors and reviewers for taking time out of their busy schedules to review our manuscripts. Thanks to the editors and all reviewers for their valuable comments, which we use to improve the quality of our manuscripts. The editor's and reviewer's comments were crucial to us, and we have carefully considered and incorporated them into the revision of the manuscript. In order to clearly show our response and changes to the manuscript, we have used the following format: reviewers' comments are listed in italics and numbered as necessary, our response is in regular font, and changes and additions to the manuscript are highlighted in blue. Once again, I would like to thank the editors and reviewers for their excellent guidance and support.

Comments marked in the manuscript by the editors and reviewers：

***Reviewer #1:***

1. *The title refers to SMEs, but the CCS case study is more typical of large enterprises. Please clarify the scope or adjust the title accordingly.*

Thank you for your suggestion, it is very helpful for us to improve the manuscript. We will adjust the title of the paper to “Research on Emission Reduction Investment Strategies for Low Carbon Technology Enterprises” to match the title of the pape.

**Changes Made:** According to this comment, the changes have been highlighted in blue in the title section of the paper.

2. *Some sections are mathematically dense and may be hard to follow for broader audiences. A simplified summary or visual diagram of the decision-making process would improve accessibility.*

Thank you for your suggestions, it is very helpful for us to improve the manuscript. We have added a simplified summary in the mathematically intensive section to improve accessibility.

**Changes Made:** According to this comment, the changes have been highlighted in blue in the Construction of composite real option model for low carbon technology projects and Construction of fuzzy composite real option model for low carbon technology projects sections of the manuscript.

3. *Consider adding a table to define variables such as η, θ, λ, and ε for clarity.*

Thank you for your suggestion, it is very helpful for us to improve the manuscript. We have added a table to define variables.

**Changes Made:** According to this comment, the changes have been highlighted in blue in the Problem description and basic assumptions section.

4*. The literature review is comprehensive but could benefit from a structured comparison table summarizing key modeling approaches (NPV, real options, fuzzy models, etc.).*

Thank you for your suggestion, it is very helpful for us to improve the manuscript. We have added a structured comparison table summarizing key modeling methods.

**Changes Made:** According to this comment, the changes have been highlighted in blue in the Literature review section.

5. *In the simulation setup, it would be helpful to provide more details on parameter values, modeling assumptions, and tools used. So, it can improve transparency to support reproducibility.*

Thank you very much for your suggestion, it is very helpful for us to improve the manuscript. This method can indeed improve transparency to support reproducibility. Therefore, in the simulation setup, we provide details about parameter values and tool usage.

**Changes Made:** According to this comment, we have modified and updated the Analysis of numerical examples section and highlighted it in blue.

6. *Please ensure all numerical data behind charts or simulations are either included in supplementary files or clearly marked in the manuscript,*

Thank you very much for your suggestion, it is very helpful for us to improve the manuscript. We clearly marked all numerical data behind the charts or simulations in the manuscript. And the source code of the simulation Figures is provided in the folder Fig1-Fig4

**Changes Made:** According to this comment, the changes have been highlighted in blue in the Parameter explanation section.

7. *Assumption 3: Please justify the use of exponential decay in O&M costs with supporting literature or data from CCS or related technologies.*

Thank you for your suggestions, it is very helpful for us to improve the manuscript. We referred to references [34] and [35] to demonstrate the rationality of using exponential decay in operational costs.

34. Steffen, Bjarne, et al. Experience curves for operations and maintenance costs of renewable energy technologies. Joule*.* 2020; 10: 359-375. doi: 10.1016/j.joule.2019.11.012

35. Gaëtan Frusque, Daniel Mitchell, Jamie Blanche, David Flynn, Olga Fink, Non-contact sensing for anomaly detection in wind turbine blades: A focus-SVDD with complex-valued auto-encoder approach. Mechanical Systems and Signal Processing. 2024; 208: 111022. https://doi.org/10.1016/j.ymssp.2023.111022.

**Changes Made:** According to this comment, relevant changes are highlighted in blue in the Problem description and basic assumptions section.

8. *Assumption 5: The linear link between emission reduction and consumer willingness to pay is likely too simplistic. Consider discussing nonlinear effects influenced by awareness, branding, or segmentation.*

Thank you for your suggestions, it is very helpful for us to improve the manuscript. We refer to reference [36] and further characterize the relationship between emissions reduction and consumer willingness to pay, based on consumers' preference for low-carbon.

36. Yuan H, Zhang L, Cao B B, et al. Optimizing traceability scheme in a fresh product supply chain considering product compe-tition in blockchain era, Expert Syst Appl. 2024; 258: 125127. https://doi.org/10.1016/j.eswa.2024.125127

**Changes Made:** According to this comment, relevant changes are highlighted in blue in the Problem description and basic assumptions section.

9. *Assumption 7: The focus on a one-time subsidy omits other common forms of support. Please justify this choice—was it due to data constraints, regional policy relevance, or model simplicity?*

Thank you for your suggestions, it is very helpful for us to improve the manuscript. We referred to reference [37, 38], and in order to simplify the model, this choice is reasonable.

37. Li, Bin, Timing Decision of Low-Carbon Technology Investment Adoption by High Energy Consuming Enterprises under Carbon Trading and Subsidies, Journal of Environmental and Public Health. 2022; 8: 9848994. https://doi.org/10.1155/2022/9848994

38. Hoda Talebian, Omar E. Herrera, Walter Mérida, Policy effectiveness on emissions and cost reduction for hydrogen supply chains: The case for British Columbia, International Journal of Hydrogen Energy. 2021; 46: 998-1011. https://doi.org/10.1016/j.ijhydene.2020.09.190.

**Changes Made:** According to this comment, relevant changes are highlighted in blue in the Problem description and basic assumptions section.

***Reviewer #2:***

1*. As the author explains the weaknesses of several conventional methods, the author needs to explain several fundamental weaknesses of the proposed plan in terms of context of use, time, and environment. Besides, the authors can explore more about the advantages and disadvantages of the proposed method*

Thank you for your suggestions, it is very helpful for us to improve the manuscript. We explained several basic drawbacks of the proposed plan from the perspectives of methodology, time, and environment. In addition, we can further explore the advantages and disadvantages of the proposed method.

**Changes Made:** According to this comment, the relevant changes are highlighted in blue in the Research Prospects section.

2. *All* *figures still need to be improved to make it easier to observe and read.*

Thank you for your suggestions, it is very helpful for us to improve the manuscript. We further optimized all the figures to make them easier to observe and read.

**Changes Made:** According to this comment, corresponding changes have been highlighted in blue in the Sensitivity analysis section.

3. *The paper is too long. The writing can be made shorter, more concise, and clearer.*

Thank you for your suggestions, it is very helpful for us to improve the manuscript. We further polished the paper, making it shorter, more concise, and clearer.

**Changes Made:** According to this comment, corresponding changes have been highlighted in blue in the Model construction and analysis of optimal investment timing, Analysis of numerical examples, Model expansion and Conclusion sections of the manuscript.

4. *Further, the author can address some potential detailed future research issues in the conclusion section.*

Thank you for your suggestions, it is very helpful for us to improve the manuscript. We have proposed some potential detailed future research questions in the conclusion section.

**Changes Made:** According to this comment, corresponding changes have been highlighted in blue in the Conclusion section.

***Additional Editor Comments:***

1. *Please ensure that your manuscript meets PLOS ONE's style requirements, including those for file naming.*

Thank you for your suggestions. We have modified the manuscript format to comply with the style requirements of PLOS ONE, including file naming requirements.

2. *Please note that PLOS ONE has specific guidelines on code sharing for submissions in which author-generated code underpins the findings in the manuscript. In these cases, we expect all author-generated code to be made available without restrictions upon publication of the work. Please review our guidelines at https://journals.plos.org/plosone/s/materials-and-software-sharing#loc-sharing-code and ensure that your code is shared in a way that follows best practice and facilitates reproducibility and reuse.*

Thank you for your suggestions. The code we generated has been placed in the fig_code file.

3. *Thank you for stating the following in your Competing Interests section: no.*

*Please complete your Competing Interests on the online submission form to state any Competing Interests. If you have no competing interests, please state "The authors have declared that no competing interests exist.", as detailed online in our guide for authors at http://journals.plos.org/plosone/s/submit-now*

*This information should be included in your cover letter; we will change the online submission form on your behalf.*

Thank you for your suggestions. We have declared in the manuscript that there are no competing interests.

4. *We note that your Data Availability Statement is currently as follows: All relevant data are included in the manuscript and its supporting information files.*

Thank you for your suggestions. The manuscript we submitted contains all the raw data required for our research results. And provided the source code of all figures in the fig_code file.

5. *Please amend your list of authors on the manuscript to ensure that each author is linked to an affiliation. Authors’ affiliations should reflect the institution where the work was done (if authors moved subsequently, you can also list the new affiliation stating “current affiliation:….” as necessary).*

Thank you for your suggestions. All authors’ affiliations are consistent with the original manuscript and do not require any modifications.

6. *Please ensure that you refer to Figure 2, 3, and 4, in your text as, if accepted, production will need this reference to link the reader to the figure.*

Thank you for your suggestions. We have cited Figures 2, 3, and 4 in the text.

7. *We note you have included a table to which you do not refer in the text of your manuscript. Please ensure that you refer to Table 1, 2, and 3, in your text; if accepted, production will need this reference to link the reader to the Table.*

Thank you for your suggestions. We have cited Tables 1, 2, and 3 in the main text, and added Tables 4, 5, and 6. The above tables have been cited in the text.
