# Supplementary material for: Research on emission reduction investment strategies for low carbon technology enterprises
Source: PLoS One. 2025 Oct 27;20(10):e0324669. doi: 10.1371/journal.pone.0324669 (PMC12558561; doi:10.1371/journal.pone.0324669)
Supplement: Attachment — Submitted filename: Response_to_Reviewers_auresp_2.docx [file pone.0324669.s008.docx]

We sincerely thank the editors and reviewers for taking time out of their busy schedules to review our manuscripts. Thanks to the editors and all reviewers for their valuable comments, which we use to improve the quality of our manuscripts. The editor's and reviewer's comments were crucial to us, and we have carefully considered and incorporated them into the revision of the manuscript. In order to clearly show our response and changes to the manuscript, we have used the following format: reviewers' comments are listed in italics and numbered as necessary, our response is in regular font, and changes and additions to the manuscript are highlighted in blue. Once again, I would like to thank the editors and reviewers for their excellent guidance and support.

Comments marked in the manuscript by the editors and reviewers：

***Reviewer:***

1*. If the authors have adequately addressed your comments raised in a previous round of review and you feel that this manuscript is now acceptable for publication, you may indicate that here to bypass the “Comments to the Author” section, enter your conflict of interest statement in the “Confidential to Editor” section, and submit your "Accept" recommendation.*

*Reviewer #1: All comments have been addressed*

*Reviewer #2: All comments have been addressed*

Thank you for your affirmation. We will continue to improve the manuscript.

*2. Is the manuscript technically sound, and do the data support the conclusions?*

*The manuscript must describe a technically sound piece of scientific research with data that supports the conclusions. Experiments must have been conducted rigorously, with appropriate controls, replication, and sample sizes. The conclusions must be drawn appropriately based on the data presented.*

*Reviewer #1: Yes*

*Reviewer #2: Yes*

Thank you for your affirmation. We will continue to improve the manuscript.

*3. Has the statistical analysis been performed appropriately and rigorously?*

*Reviewer #1: Yes*

Thank you for your affirmation. We will continue to improve the manuscript.

*Reviewer #2: N/A*

Thank you for your suggestion, it is very helpful for us to improve the manuscript. We have cited specific literature to set the parameters of the paper more reasonably, ensuring that the statistical analysis is more appropriate and reasonable.

**Changes Made:** According to this comment, the changes have been highlighted in blue in the Parameter explanation section of the paper.

*4. Have the authors made all data underlying the findings in their manuscript fully available?*

*The PLOS Data policy requires authors to make all data underlying the findings described in their manuscript fully available without restriction, with rare exception (please refer to the Data Availability Statement in the manuscript PDF file). The data should be provided as part of the manuscript or its supporting information, or deposited to a public repository. For example, in addition to summary statistics, the data points behind means, medians and variance measures should be available. If there are restrictions on publicly sharing data—e.g. participant privacy or use of data from a third party—those must be specified.*

*Reviewer #1: Yes*

*Reviewer #2: Yes*

Thank you for your affirmation. We will continue to improve the manuscript.

*5. Is the manuscript presented in an intelligible fashion and written in standard English?*

*PLOS ONE does not copyedit accepted manuscripts, so the language in submitted articles must be clear, correct, and unambiguous. Any typographical or grammatical errors should be corrected at revision, so please note any specific errors here.*

*Reviewer #1: Yes*

*Reviewer #2: Yes*

Thank you for your affirmation. We will continue to improve the manuscript.

*6. Review Comments to the Author*

*Please use the space provided to explain your answers to the questions above. You may also include additional comments for the author, including concerns about dual publication, research ethics, or publication ethics. (Please upload your review as an attachment if it exceeds 20,000 characters)*

*Reviewer #1: (No Response)*

*Reviewer #2: All comments have been addressed, particularly related to some potential detailed future research issues.*

Thank you for your affirmation. We will continue to improve the manuscript.

***Additional Editor Comments:***

*Thank you for submitting your manuscript to PLOS ONE. After careful consideration, we feel that it has merit but does not fully meet PLOS ONE’s publication criteria as it currently stands. Therefore, we invite you to submit a revised version of the manuscript that addresses the points raised during the review process.*

Thank you for your suggestions. We have further revised the paper based on the comments of the reviewers. In addition, we have modified the reference format of the paper according to the journal's formatting requirements and inserted Figure into the paper instead of displaying it in Supporting Information.

**Changes Made:** According to this comment, the changes have been highlighted in blue in Parameter explanation, Sensitivity analysis, References and Supporting information sections of the paper.
